# Mesenchymal Stem Cells in Combination with Scaffolds for Bone Tissue Engineering

**DOI:** 10.3390/ma4101793

**Published:** 2011-10-11

**Authors:** Laeticia Nassif, Marwan El Sabban

**Affiliations:** 1 Division of Orthodontics, American University of Beirut, P.O.Box 11-0236, Riad El-Solh, Beirut 1107-2020, Lebanon; E-Mail: laeticianassif@hotmail.com; 2 Department of Anatomy, Cell Biology and Physiological Sciences, Faculty of Medecine, American University of Beirut, P.O.Box 11-0236, Riad El-Solh, Beirut 1107-2020, Lebanon

**Keywords:** stem cells, bone grafts, biomaterial, tissue engineering

## Abstract

This article reviews past and current strategies of the use of bone graft substitutes along with the future biologic alternatives that can enhance the functional capabilities of those grafts. Many of these bone graft substitute alternatives include ceramic-based, allograft-based, factor-based and polymer-based whereas others are cell-based. The ways of achieving the goal of tissue engineering using stem cells and their lineage to regenerate tissue have been detailed with regard to both the generation of sufficient vascular invasion of the tissue to improve oxygen and nutrient supply, and the development of innovative physical/chemical stimuli to induce bone formation with the proper biomaterial to carry the cells. It is imperative to integrate basic polymer science with molecular biology and stem cell biology, in the design of new materials that perform very sophisticated signaling needed for integration and function.

## 1. Introduction

Bone grafts are used by orthopedic surgeons, neurosurgeons, craniofacial surgeons, and periodontists. Autografts are the golden standard for this procedure because they possess all of the characteristics necessary for new bone growth namely, osteoconductivity, osteogenicity, and osteoinductivity. It has been well documented that there are limitations and complications in the use of autografts, including the limited availability and the associated chronic donor site pain. 

Allografts are an alternative to autografts and are recovered from living or deceased donors. Allograft tissue is treated with tissue freezing, freeze-drying, electron beam radiation, ethylene oxide, or gamma irradiation but even with such sterilization techniques, the risk of disease transmission from donor to recipient cannot be absolutely eliminated. Nucleic acid testing (NAT) to screen for HIV and HCV is an efficient tool to eliminate this risk, since it uses a highly sensitive polymerase chain reaction test to look for genetic material of viruses such as HIV and HCV. This screening increases the safety margin for allograft use by decreasing the “window period” not detectable by traditional antibiotic testing [1,2,3]. The probability of donor viremia would be reduced to 1 in 315,000 for HIV, 1 in 385,000 for HBV and 1 in 500,000 for HCV. The prevalence of HIV, HBV, HCV and HTLV although low, is higher among musculoskeletal tissue donors than among first-time blood donors. The risks associated with musculoskeletal donation could be reduced with NAT, although further cost analysis would be required prior to its implementation [4]. 

Since 25 May 2005, all manufactured or processed allograft tissues have been subject to regulation to ensure that there exists a comprehensive risk base system for regulating human cells, tissues, and cellular and tissue-based products. The AATB is a voluntary professional accrediting organization for the tissue banking industry. It is a scientific non-profit peer group organization that promotes high-quality transplantable human tissues. An AATB-accredited tissue bank undergoes onsite inspections every 3 years and, as a processor, must show that its procedures comply with AATB standards [5]. The last documented case for disease transmission was in 2005 [6].

On the other hand, processing may impact on biologic, structural integrity and mechanical properties in addition to the immune reaction going from an acute to a chronic reaction, potentially affecting incorporation and longevity of the graft [7,8,9,10]. Another disadvantage for allografts is the increased cost which depends on the type of graft used and the procedure [11].

Thus, there is a tremendous need and responsibility to develop biologic alternatives that will enhance the functional capabilities of the bone graft substitute, and potentially reduce or eliminate the need for autograft. This article reviews past and current strategies of the use of bone graft substitutes and the future directions of research. Many of these bone graft substitute alternatives use a variety of materials, including natural and synthetic polymers, ceramics and composites, whereas others have incorporated factor- and cell-based strategies that are used either alone or in combination with other materials. 

Laurencin *et al.* (2006) [12], have suggested a classification scheme for material-based categories of bone graft substitute groups. Many are formed from composites of one or more types of material. These products vary considerably in chemical composition, structural strength, and resorption or remodeling rates. Understanding these differences is important in selecting a bone graft substitute with the properties desired for a specific clinical situation [13].

**Table 1:** Classification for material-based categories of bone graft substitute groups.

Class	Description
1.1 Ceramic based	Includes calcium phosphate, calcium sulfate, and bio-glass, used alone or in combination
1.2 Allograft based	Allograft bone, used alone or in combination with other materials
1.3 Factor based	Natural and recombinant growth factors, used alone or in combination with other materials
1.4 Polymer based	Both degradable and non-degradable polymers, used alone or in combination with other materials
1.5 Cell based	Cells used to generate new tissue alone or seeded onto a support matrix

### 1.1. Ceramic-Based Bone Graft Substitute 

Currently available bone graft substitutes involve ceramics, either alone or in combination with another material. These include calcium sulfate, bioactive glass, and calcium phosphate. The primary inorganic component of bone is calcium hydroxyapatite (HA), a subset of the calcium phosphate group. The most commonly used calcium phosphate ceramics are HA and tricalcium phosphate (TCP), used in the form of implant coatings and defect fillers. HA and TCP coating of metal surfaces enhance ingrowth and direct bonding of bone to porous surface [14,15,16]. These materials are highly crystalline, bio-inert ceramics, which are not moldable intraoperatively. Like HA, TCP is bio-absorbable and biocompatible. The solubility and degradation profile of HA and tricalcium phosphates varies between a very stable, almost insoluble appearance (100% HA) to a fast degradation and loss of structure (100% α-tricalciumphosphate, TCP) [17]. For optimization of the properties of the composite materials for cell seeding and tissue engineering applications, several studies of different ratios of HA and TCP have been conducted and reported [18]. 

In addition to HA and TCP, glass-ceramic macroporous scaffolds have been developed using a polyurethane sponge template and bioactive glass powders and have proved to be good candidates as scaffolds for bone-tissue engineering, in terms of pore-size distribution, pore interconnection, surface roughness, and both bioactivity and biocompatibility [19].

### 1.2. Allograft-Based Bone Graft Substitute

Contained in the extracellular matrix of bone tissue is a combination of bone growth factors, proteins, and other bioactive materials necessary for osteoinduction and, ultimately, successful bone healing. To capitalize on this cocktail of proteins, the desired factors and proteins are removed from the mineralized tissue by using a demineralizing agent such as hydrochloric acid. The mineral content of the bone is degraded, and the osteoinductive agents remain in a de-mineralized bone matrix (DBM). The osteoinductive capacity of DBM has been well established [20]. The components of the bone that remain behind include the non-collagenous proteins and bone osteoinductive growth factors, the most significant of which is BMP and type I collagen. DBM provides no structural strength, and its primary use is in a structurally stable environment. A carrier may be added to DBM to improve its handling characteristics and mechanical properties. DBM obtained from allogeneic human cortical bone shows a variable efficacy and osteoinductive index.

Collagen lyophilizates are sterile acellular extracts of bone matrix .They contain mainly collagen type I and associated proteins and growth factors involved in the cascade of bone formation. The preparatory methods are similar to those originally used by Urist *et al*. in 1971. Isolation of bone morphogenetic protein, the non-collagenous components, has been presumed to include some growth factors such as VEGF, TGFβ-1, TGFβ-2 and BMP-3. *In vitro* studies [21], as well as *in vivo* studies [22,23,24] have proven materials to be osteoinductive.

### 1.3. Factor-Based Bone Graft Substitute

The factors and proteins that exist in bone are responsible for regulating cellular activity. Growth factors bind to receptors on cell surfaces, stimulating the intracellular environment to act. Generally, this activity translates to a protein kinase that induces a series of events resulting in the transcription of messenger ribonucleic acid (mRNA) and, ultimately, into the formation of a protein to be used intracellularly or extracellularly. The combination and simultaneous activity of many factors result in the controlled production and resorption of bone. These factors, residing in the extracellular matrix of bone, include Transforming Growth Factor beta TGF-ß TGF-beta, insulin like growth factors I and II, platelet-derived growth factors PDGF, fibroblast growth factor FGF, osteogenic proteins (OP 1) and bone morphogenic proteins BMPs [25,26,27,28,29,30]. BMPs form a unique group of proteins within the TGF-ß superfamily of genes and have pivotal roles in the regulation of bone induction, maintenance and repair. They act through an autocrine or paracrine mechanism by binding to cell surface receptors and initiating a sequence of downstream events that have effects on various cell types. Differentiation of osteoprogenitor mesenchymal cells and up-regulation of osteoblastic features occur under the influence of cytokines and growth factors that are expressed with the direct or indirect guidance of BMPs acting at the transcriptional level or higher. The Smads family of proteins has been identified as the downstream propagator of BMP signals, whereas hedgehog genes are possible modulators of BMP expression. The inflammatory response observed during wound repair and fracture healing, results in by-products that interact with BMPs and affect their biologic potential. Additive, negative or synergistic effects are observed when homodimeric or heterodimeric forms of BMPs interact with BMP receptors. Storage within the bone matrix allows for their involvement in the modeling/remodeling process by mediating the coupling of osteoblasts and osteoclasts. Micro-environmental conditions, dose, possible carrier materials and geometrical parameters of delivery matrix are critical determinants of the pharmacokinetics of BMP action and the biologic outcome during wound repair. Because of their osteogenic potential, BMPs are of tremendous interest as therapeutic agents for healing fractures of bones, preventing osteoporosis, treating periodontal defects and enhancing bone formation into alloplastic materials implanted in bone [31]. Like BMPs, OPs have also proved to enhance bone repair [32].

### 1.4. Polymer-Based Bone Graft Substitute

Currently used polymers can be loosely divided into natural polymers and synthetic polymers. These, in turn, can be divided further into degradable and nondegradable types. Polylactic and polyglycolic acid polymers have the advantage of being integrated with growth factors, drugs, and other compounds to create multiphase delivery systems [33,34]. Type I collagen has a structure that is conducive in promoting mineral deposition and it binds the noncollagenous matrix proteins, which initiate and control mineralization by themselves. Mineralized collagen sponges made of Type I collagen fibres coated with hydroxyapatite when coupled with bone morphogenetic proteins, and osteoprogenitor precursors enhance incorporation of grafts significantly. As biodegradable scaffolding polymers, collagen-based sponges, demineralized bone matrix, poly-L-lactic acid, poly-L-glycolic acid polymers and gelatin sponges were demonstrated to have several benefits [35]. Their drawbacks include immunogenicity, going from an acute immune reaction to a chronic one and weak mechanical strength. Chitosan was developed later. Chitin and chitosan represent a family of biopolymers, made up of b(1-4)-linked *N*-acetyl-d-glucosamine and d-glucosamine subunits. Chitin can be found widely in the exoskeletons of arthropods, shells of crustaceans, and the cuticles of insects [36]. 

Chitosan is produced industrially by alkaline hydrolysis of chitin *N*-Acetyl-glucosamine which is present in glycosaminoglycans of the extracellular matrix (ECM) and of the cell surface, usually linked to core proteins as proteoglycans. Chitosan can thus provide a non-protein matrix for 3D cell in-growth, structurally similar to extracellular proteoglycans [37]. The soluble form of chitosan can have a degree of acetylation (DA) which is, by definition, the molar fraction of *N*-acetylated units between 0% and about 60%, the upper limit, and depends on parameters such as processing conditions, molar mass and solvent characteristics. The (DA), together with the molecular weight, are the most important parameters used to characterize chitosan. The DA is a structural parameter influencing solubility, crystallinity, charge density, and also the susceptibility to enzymatic degradation, with higher DAs leading to faster biodegradation rates [38]. Three sterilization methods (immersion in 100% ethanol, exposure to ethylene oxide, or gamma radiation) are used and are suitable for use on chitosan membranes. Despite the fact that gamma radiation is one of the most common agents employed for biomaterial sterilization, it causes modifications to the chitosan membrane structure, which were observed by color change of the samples. The use of 100% ethanol for 48 h as a sterilizing agent showed adequate results, however, it is limited to small-scale use [39]. Chitosan is biodegradable biocompatible, osteoconductive and able to enhance osteogenesis and angiogenic activity [40,41,42,43,44] and inhibit fibrous tissue invasion which prevents new bone tissue generation within the scaffold [45]. The association of chitosan with calcium phosphates and biologically active biomolecules was also reported, in an attempt to improve its mechanical properties, osteoconduction and bone regeneration induction [46].

### 1.5. Cell-Based Bone Graft Substitute

Nowadays, osteoblast transplantation using polymer scaffolds is a promising strategy that involves the use of polymer-cell constructs composed of osteogenic cells that can be obtained from the host and grown in the carrier *in vitro* [47]. Subsequently, the osteogenic tissue scaffold constructs can be grafted back into the host to regenerate bone. Current concepts of bone regenerative medicine emphasize the need for new biocompatible and bioresorbable filling materials, structurally mimicking the extracellular matrix, capable of acting as vehicles for autologous osteoprogenitor cells and/or cell signaling molecules. Among other features, scaffolds are expected to allow loading, division, and retention of progenitor cells, support rapid vascular ingrowth and encourage osteoconduction with host bone [48]. On the other hand, the transplanted cells may secrete a new matrix as well as the factors necessary for bone tissue growth and ingrowth while the polymer matrix gradually degrades [49]. At present, chitosan is being investigated as a temporary support for the growth of a number of anchorage-dependent cells, including osteoblasts, for tissue engineering applications [50,51]. Recently, the effect of the DA of chitosan on the osteogenic differentiation of bone marrow stromal cells (BMSCs) was reported in a 2D culture. An investigation of culturing osteoblast cell-sponge constructs [52] demonstrated that chitosan sponges support the differentiation of seeded osteoblastic cells as well as their proliferation. These results show that Chitosan sponges can be used as effective scaffolding materials for tissue engineered bone formation in vitro and in vivo. Chitosan’s cationic nature also allows for pH-dependent electrostatic interactions with anionic glycosaminoglycans, proteoglycans and other negatively charged species. These ionic interactions may serve as a mechanism for retaining or accumulating these molecules within the scaffold. Since a large family of growth factors and cytokines are bound and modulated by GAGs (in particular heparin and heparan sulfate), a scaffold incorporating a Chitosan-GAG complex may provide a means of retaining and concentrating desirable factors secreted by colonizing cells. Such a system may even be capable of recruiting desirable growth factors from surrounding tissue and or enhancing migration and differentiation of specific types of progenitor cells [53]. 

## 2. Approaches for Achieving the Goal of Tissue Engineering

### 2.1. Using Stem Cells and Their Lineage to Regenerate Tissue

Since Friedenstein and colleagues first publications in the 1980s [54,55], it has been known that mesenchymal stem cells (MSCs) can be used to engineer mesenchymal tissues, such as bone and cartilage and that the most abundant source of MSCs, which has a high proliferative ability and great capacity for differentiation is bone marrow. Therefore, scientists worldwide are working to provide the right carrier and the appropriate set of cells that, once re-transplanted, will ensure bone repair.

### 2.2. Providing Sufficient Vascular Supply to Improve Oxygen and Nutrient Supply

Lack of sufficient vascular supply, resulting in immediate cell death after implantation, is generally thought to be the cause of failure of BTE in patients [56]. The success of bone tissue engineering in ectopic rodent models is explained by the far more favorable biological environment for implanted cells. Vessel growth is stimulated immediately after application of the cell-based graft [57]. Often only small samples are subcutaneously implanted, which are then in direct contact with the surrounding well-vascularized tissues. These shorten the diffusion depth, allowing the seeded MSCs to be optimally supplied by oxygen and nutrients. In addition, osseous defects in rodents are attractive sites for reconstruction. Defect sizes do not exceed the maximum distant depth of 5 mm, thus, allowing sufficient influx of oxygen and nutrition [58]. 

A second method, simply bypasses the problems linked to orthotopic bone formation by creating an engineered bone construct in a muscular environment (ectopic bone formation). Warnke and his colleagues in 2004 [59], reported a successful reconstruction of an extended mandibular discontinuity defect by growth of a custom bone transplant inside the latissimus dorsi muscle of an adult male patient. The transplant was then moved as a pedicle bone-muscle flap to repair the mandibular defect. 

As a third approach, postponing the application of human MSCs for a few days after applying the scaffold would be a viable option. Immediately after implantation of the scaffold, a hematoma is formed [60]. On the third or fourth day, during the chronic inflammation phase, blood vessels and fibroblasts proliferate in the fibrin clot, thus forming granulation tissue [61]. By injecting the culture expanded MSCs at this point in time, it ensures that the new blood vessels are already invading the hematoma, thereby guaranteeing a sufficient supply of oxygen. However, an alternative direction for bone tissue engineering can be through the use of appropriate scaffolds that attract the patient’s own stem cells post-implantation [62].

### 2.3. Developing Innovative Physical/Chemical Stimuli to Induce Bone Formation

It has long been known that a bone matrix contains an active ingredient, bone morphogenetic protein (BMP), that can hasten the repair process or induce bone formation outside of the skeleton when introduced in combination with an appropriate scaffold [63]. The induced bone can ultimately disappear owing to the eventual degradation of the BMP. However a potential drawback of this approach is that, high, supra-physiologic concentrations are needed to obtain the desired osteoinductive effect, with possible related side effects and high costs. For this reason, efforts are under way to use molecular techniques to enhance BMP production by cells before their use in tissue engineering. 

### 2.4. Developing the Proper Biomaterial to Carry the Cells

Scaffolds are constructs, which are used as a support structure allowing the cells to adhere, proliferate and differentiate to form a healthy bone tissue for restoring the functionality. The scaffolds can be classified into two different categories: permanent and temporary implants. Permanent scaffolds retain their shape and strength through the process of regeneration and repair of the organ while the temporary scaffolds degrade over a period of time with the regeneration of the organ or tissue. Applications such as oral and maxillofacial surgery rely on the hard permanent scaffolds such as titanium-based scaffold for reconstruction or regeneration of the tissue or organ. Titanium and its alloys have been widely used as scaffolds for maxillofacial and craniofacial reconstructions over the past two decades. Titanium meshes with porous network in combination with hydroxyapatite provide the necessary bioactivity to facilitate osteoconduction. This method has been found to be successful and a viable alternative to autograft and allografting methods [64]. 

The structure of a scaffold plays an important role in guiding tissue development. For most tissues, the key requirement that can be defined at present is that interconnected porosity of larger dimensions than the cells is required or desired. Internal pore architecture is necessary to maximize nutrient diffusion, interstitial fluid [65] and blood flow [66] to control cell growth and function [67]. It is also necessary to manipulate tissue differentiation, and to optimize scaffold mechanical function and generated tissue mechanical properties [68,69]. This is based on three-dimensional interconnections between the lacunae in the bones that provide intercellular communication. Porosity with interconnectivity is the most essential prerequisite for osteoconduction. Although there are alternative views, the consensus of research indicates that the requisite pore size for bone ingrowth into porous implants is 100 to 500 µm, and the interconnections must be larger than 100 µm [70]. 

On the other hand, the highest probability for substantive cell movement through pores was observed for an intermediate pore diameter, rather than the largest pore diameter, which exceeded the cell diameter. The relationships between migration speed, displacement, and total path length were found to depend strongly on pore diameter. This dependence was attributed to convolution of pore diameter and void chamber diameter, yielding different geometric environments experienced by the cells within [71]. 

## 3. Conclusions

Research has been focused on the development of scaffolds, to be made of biodegradable materials for most tissue engineering applications. One of the most important changes spreading in the field is the awareness of the strong need to integrate basic polymer science with molecular cell biology and stem cell biology in the design of new materials that carry out very sophisticated signaling needs.
